# Snake Venomics and Antivenomics of *Bothrops diporus*, a Medically Important Pitviper in Northeastern Argentina

**DOI:** 10.3390/toxins8010009

**Published:** 2015-12-25

**Authors:** Carolina Gay, Libia Sanz, Juan J. Calvete, Davinia Pla

**Affiliations:** 1 Facultad de Ciencias Exactas y Naturales y Agrimensura, Universidad Nacional del Nordeste, Avenida Libertad 5470, 3400 Corrientes, Argentina; claudiacarolinagay@yahoo.com.ar; 2 Instituto de Biomedicina de Valencia, CSIC, Jaime Roig 11, 46010 Valencia, Spain; libia.sanz@ibv.csic.es

**Keywords:** *Bothrops diporus* venom, venomics, snake venom proteome, antivenomics, mass spectrometry

## Abstract

Snake species within genus *Bothrops* are responsible for more than 80% of the snakebites occurring in South America. The species that cause most envenomings in Argentina, *B. diporus*, is widely distributed throughout the country, but principally found in the Northeast, the region with the highest rates of snakebites. The venom proteome of this medically relevant snake was unveiled using a venomic approach. It comprises toxins belonging to fourteen protein families, being dominated by PI- and PIII-SVMPs, PLA_2_ molecules, BPP-like peptides, L-amino acid oxidase and serine proteinases. This toxin profile largely explains the characteristic pathophysiological effects of bothropic snakebites observed in patients envenomed by *B. diporus*. Antivenomic analysis of the SAB antivenom (Instituto Vital Brazil) against the venom of *B. diporus* showed that this pentabothropic antivenom efficiently recognized all the venom proteins and exhibited poor affinity towards the small peptide (BPPs and tripeptide inhibitors of PIII-SVMPs) components of the venom.

## 1. Introduction

*Bothrops diporus* [1], commonly known as “yarará chica”, is a venomous terrestrial lancehead pit viper endemic to South America. *B. diporus* was initially classified as one of the twelve subspecies of the *Bothrops neuwiedi* complex [2]. Revisions of the systematics within the *neuwiedi* group elevated five subspecies, including *B. n. diporus*, to the species level [3]. Recently, Fenwick and co-workers have proposed an alternative taxonomic arrangement [4]. These authors proposed a new genus, *Bothropoides* (derived from the Greek *bothros* and *ops* referring to the facial pit, and the term *oides* meaning “similar to”) to include *B. alcatraz*, *B*. *diporus*, *B*. *erythromelas*, *B*. *insularis*, *B*. *jararaca*, *B*. *lutzi*, *B*. *mattogrossensis*, *B*. *neuwiedi*, *B*. *pubescens*, and *B*. *pauloensis.* Taxonomical revisions may have an important impact in the medical area because a correct identification of the species responsible for snakebite accidents in a given area is of applied importance for the production and efficient use of the antivenoms. In addition, an accurate taxonomy will enlighten research in comparative biology, trait evolution, historical biogeography, and other fields. In this respect, Carrasco *et al.* [5] highlighted the incongruence between their analyses using morphological, ecological and molecular information of all species of the *B. neuwiedi* group, and the classification proposed by Fenwick and co-workers [4]. The demonstration that *B. neuwiedi* is a highly supported monophyletic group, invalidated Fenwick *et al.*’s proposal of splitting *Bothrops* in three new genera: *Bothropoides* (*B. neuwiedi* group and *B. jararaca* group), *Rhinocerophis* (*B. alternatus* group) and *Bothrops* sensu strict (*B. atrox* group).

*B. diporus*, a relatively small, medium build (adult males average 60–70 cm in total length, and adult females may grow to as much as 100–110 cm) can be found in semitropical deciduous forests, wet palm-grasslands, Chaco, *Araucaria* forests, and Pampas in Central Brazil (States of Mato Grosso do Sul, São Paulo, Paraná, Santa Catarina and northwestern Rio Grande do Sul), in extreme Southcentral Bolivia, Paraguay, and in the Argentinian provinces of La Rioja, La Pampa, Córdoba, San Luis, Mendoza, Neuquén, Catamarca, Santiago del Estero, Tucumán, Jujuy, Salta, Formosa, Chaco, Santa Fé, Entre Ríos, Corrientes and Misiones, reaching as far south as Northern Patagonia [2,6]. Due to its abundance, highly aggressive behavior, and wide geographical distribution, *B. diporus* is a major source of snakebites in Argentina, with the northeastern region representing the part of the country with the highest rate of bites by this species [7,8,9].

A study of the toxic and enzymatic activities of venoms collected from specimens of different regions of Argentina established a remarkably similar toxicity profile throughout its range [10]. No significant differences in the LD_50_ values (51.8 to 82.6 μg/mouse) were found, and the only conspicuous difference in the toxicological pattern of *B. diporus* venoms was the low-thrombin-like activity found in the sample from Formosa [10]. Despite its medical relevance, the venom of *B. diporus* is still poorly characterized. Only a few proteins have been cloned, isolated and/or biochemically or functionally characterized, including the PLA_2_ molecules, Myo-II (AFJ79209), s PLA_2_-I (AFJ79207), sPLA_2_-II (AFJ79208), svPLA_2_ (C0HJP9) [11,12,13,14,15,16], and the *C*-type lectin-like protein SL1_BOTDP (C0HJQ0). However, a detailed view of the venom proteome is still missing. To gain a deeper insight into the spectrum of medically important toxins present in the venom of *B. diporus*, we sought to define its venom proteome using a venomics approach.

The parenteral administration of antivenoms constitutes the mainstay in the therapy of snakebite envenomings. Antivenoms are produced by immunizing animals, mostly horses, with the venoms of one or several species, thus generating monospecific or polyspecific antivenoms, respectively [17]. The most widely anti-bothropic antivenom used in Argentina for the treatment of bothropic envenomings, is a F(ab′)_2_ antivenom produced by the Instituto Nacional de Producción de Biológicos (INPB, Buenos Aires, Argentina) using venom of *B. alternatus* and *B. (neuwiedi) diporus* as immunogens [18]. This antivenom efficiently neutralized lethality and all toxic activities of *B. diporus* tested [10]. Although there are a number of antivenom manufacturers in Latin America [19], they differ in their technological platforms and scales of production. Thus, there can be circumstances where the local production of an antivenom is insufficient to cover national needs and products from other countries have to be imported. Previous studies have demonstrated a high degree of cross-neutralization of antivenoms produced in several Latin American countries, although in other cases antivenoms were ineffective in the neutralization of some activities of heterologous venoms [20]. Here, we have assessed the cross-immunorecognition of the bothropic antivenom produced by Instituto Vital Brazil against the venom toxins of *B. diporus*.

## 2. Results and Discussion

### 2.1. Characterization of the Venom Proteome of B. diporus. Comparison with the Toxin Composition of Venoms from Species of the B. neuwiedi Complex

**Figure 1:**
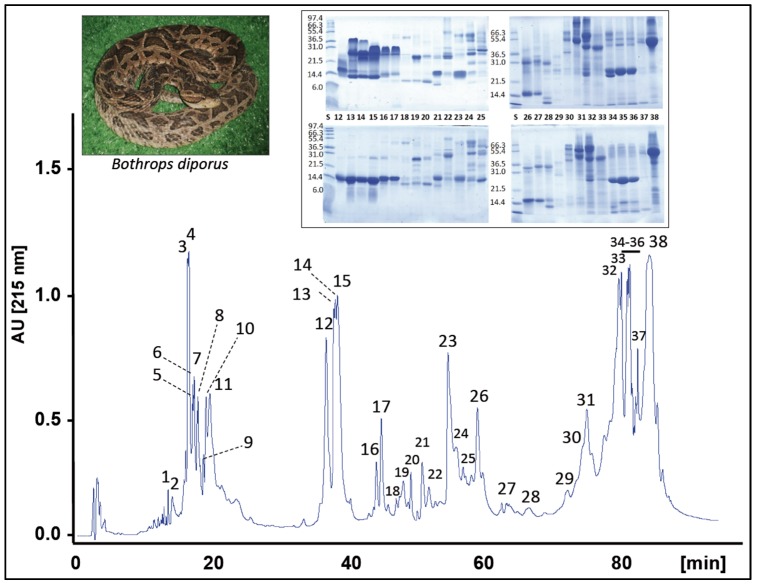
Reverse-phase HPLC separation of the venom proteins from *B. diporus*. Insert, SDS-PAGE of the isolated chromatographic fractions run under non-reduced (upper panels) and reduced (lower panels) conditions.

**Figure 2:**
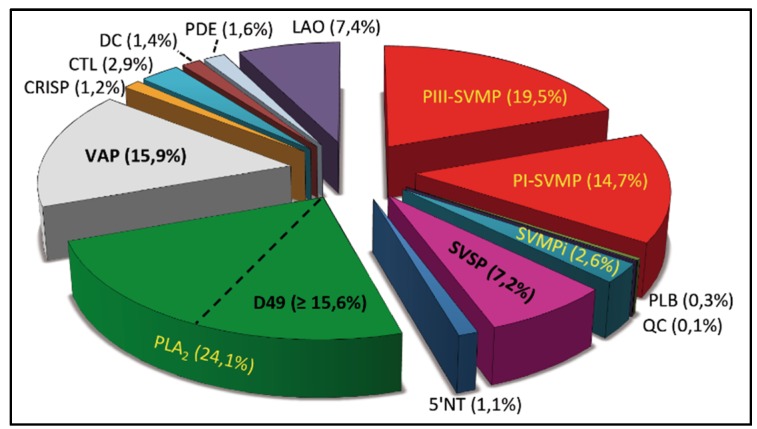
Relative protein composition (in % of the total venom proteins) of *B. diporus* venom. PIII-SVMP and PI-SVMP, snake venom metalloproteinases of class PIII and PI, respectively; SVMPi, snake venom metalloproteinase tripeptide inhibitors; PLB, phospholipase B; QC, glutaminyl cyclase; SVSP, snake venom serine proteinase; 5′NT, 5′ nucleotidase; PLA_2_, phospholipase A_2_; VAP (BPP, bradykinin-potentiating peptide and BPP-like peptides); CRISP, cysteine-rich secretory protein; CTL, *C*-type lectin-like protein; DC, disintegrin-like/cysteine-rich domains; PDE, phosphodiesterase; PLB, phospholipase B; LAO, L-amino acid oxidase.

The venom of *B. diporus* was fractionated into 38 RP-HPLC fractions (Figure 1). Each chromatographic fraction was analyzed by SDS-polyacrylamide gel electrophoresis (Figure 1, insert), and the protein bands were excised and submitted to mass spectrometric analysis [21]. The MS/MS data, listed in Supplementary Table S1, resulted in the identification of proteins and peptides belonging to 14 snake venom protein families, whose relative abundances are displayed in Figure 2.

The venom proteome of *B. diporus* is predominantly comprised of PI- and PIII-SVMPs, PLA_2_ proteins, vasoactive peptides, LAOs, and SVSPs. Each of these protein classes represents ≥7% of the total venom proteins. Similar qualitative protein family distribution has been described in venoms of other species of the *B. neuwiedi* complex, such as *B. pauloensis* [22] and *B. neuwiedi* [23], although each species differs from the other in the relative abundances of its major toxin classes: *B. pauloensis* (SVMPs, 38%; PLA_2_, 32%; VAP, 12.4%; SVSP, 10.5%), *B. neuwiedi* (SVMPs, 50%; LAO, 16.7%; SVSP, 8.8%; CTLs, 8.6%; PLA_2_, 8.4%). However, regarding *B. neuwiedi*, the venom peptidome was not investigated [22]. On the other hand, the minor venom components (e.g., those accounting for ≤3% of the total venom proteins) found in *B. diporus* venom include CTLs, tripeptide inhibitors of SVMPs (SVMPi), PDE, DC-fragments of PIII-SVMPs, CRISP, 5′-NT, PLB, and glutaminyl cyclase (QC), whereas low abundance toxin classes in *B. pauloensis* and *B. neuwiedi* venoms are, respectively, LAO (2.8%), CRISP (2.2%), Disintegrin (1.3%), *C*-type Gal-lectin (0.6%), and nerve growth factor (0.2%) and (CRISP (2%); 5′NT (1.5%); PLB (1.4%); PDE (1.2%); QC (0.8%); vascular endothelial growth factor (0.4%); and nerve growth factor (0.3%).

### 2.2. Correlations between Major Venom Proteins and Venom Toxicity

The venom of *B. diporus* is highly proteolytic, hemorrhagic and myotoxic [10,16,20,23,24,25]. Its toxin profile here reported potentially explains the local and systemic effects observed in envenomings by this species, which, as in most accidents due to species of the genus *Bothrops* [26,27], are associated with major local effects (rapid edema formation, pain, inflammation, ecchymosis, hemorrhage, and local myonecrosis, dermonecrosis and blistering) and, in moderate to severe cases, systemic manifestations (blood clotting perturbations, hypotensive shock, and kidney failure) may also develop [23,28].

Local damage is mainly caused by cytolytic PLA_2_ molecules [29,30,31] and extracellular matrix-disrupting Zn^2+^-dependent PIII-SVMPs [32,33]. The high abundance of PLA_2_ (24.1%, of which ≥65% correspond to the proteolytically active D49 class) and PIII-SVMPs (19.5%, of the total venom proteome, half of which corresponds to the 48 kDa protein *B. jararaca* jararhagin-like SVMP [P30431] eluted in fraction 38) in *B. diporus* venom proteome may contribute to the severity of local effects caused by this species. Myonecrosis, edema, inflammation, and acute muscle damage, are also widely correlated with PLA_2_ molecules [30,31]. Thus, acidic PLA_2_ catalytic isoenzymes (PI and PII) isolated from *B. diporus* venom exhibited an important edema-inducing activity but lacked myotoxicity [14]. The isotope-averaged molecular masses (13.649,9 Da and 13.621,8 Da), along with tryptic peptide sequences representing 65% of sequence coverage, identified the 14 kDa proteins recovered in RP-HPLC fraction 23 (Figure 1) as sPLA_2_-I [AFJ79207] and sPLA_2_-II [AFJ79208], respectively. These acidic secretory PLA_2_ isoenzymes account for 3.3% of the total venom proteome (Table S1). On the other hand, Geoghegan *et al.* [16] have reported a basic myotoxic K49-PLA_2_ homologue, myotoxin I, showing potent myotoxic, cytolytic, and edema-inducing activities.

When injected at relatively low doses, *B. diporus* venom induces desfibrinogenation in mice [21]. PI-SVMPs are potent proteolytic and alpha > β-fibrino(geno)lytic enzymes [34,35]. PI-SVMPs may thus contribute to the depletion of circulating clottable fibrinogen and can, in conjunction with the action of thrombin-like serine proteinases targeting coagulation factors [36,37,38], synergistically potentiate the activity of hemorrhagic PIII-SVMPs, resulting in increased incidence of systemic bleeding. *C*-type lectin-like molecules also interact with components of the human hemostatic system, affecting the blood coagulation cascade and platelet aggregation, further contributing to blood clotting perturbations caused by *B. diporus* venom [39,40].

L-amino acid oxidases, flavoenzymes that catalyze oxidative deamination of L-amino acids to form corresponding α-keto acids, hydrogen peroxide and ammonia, are widely distributed in viperid venoms [41]. Certain L-amino acid oxidases, such as *B. pauloensis* B5AR80, have been reported to induce platelet aggregation in platelet-rich plasma [42]. However, the contribution to the envenoming process of the *B. diporus* LAO eluted in RP-HPLC fraction 30, representing 7.5% of the total venom proteins, remains elusive.

The high abundance of putative vasoactive peptides (15.9% of the venom proteome) (Figure 2) is remarkable. BPP + BPP-like peptides, commonly found in the venom of many *Bothrops* and other snake species [43], include inhibitors of the angiotensin I-converting enzyme that enhance the hypotensive effect of circulating bradykinin, causing a vascular shock in the snake’s prey or victim [44,45,46]. However, BPP-like peptides lacking the *C*-terminal sequence (PXIPP) caused no potentiation of bradykinin hypotensive effect [47,48] and, despite being a major venom component of *Lachesis spp*. venoms, do not represent a serious clinical concern in the treatment of envenomings by species of this genus [49]. This evidence suggests that the PXIPP motif is crucial for the proper expression of the pharmacological activities of BPPs. Peptides ZARPPHPPIPP (RP-HPLC-4), (254.3)VNAPXNPSIPP and (237.2)ADPRAPNIPP (RP-HPLC-10), and ZGGQPTPQIPP (RP-HPLC-11), representing, respectively, 1.7%, 1.3%, and 7.1% of the venom fractions, bear this *C*-terminal sequence motif (Table S1). A related peptide, ZGGWPRPGPEIPP, isolated from “*B. neuwiedi*” venom showed bradykinin-potentiating action on isolated guinea-pig ileum and a relevant angiotensin-converting enzyme competitive inhibiting activity [50]. The pharmacological activity of *B. diporus* venom BPPs deserves future detailed investigation. BPPs typically contain an *N*-terminal 5′-oxoprolinyl (pyroglutaminyl) residue. Snake venom QC may catalyze the cyclization of *N*-terminal glutamic acid and glutamine residues [51] of BPPs and other venom components, such as PIII-SVMPs and their endogenous tripeptide inhibitors (SVMPi), ZNW, ZQW, and ZKW [52,53]. Present at millimolar concentrations [54], these low-affinity antagonists maintain SVMPs catalytically inactive in the lumen of the venom gland, and their inhibition is instantly disengaged when venom is injected into tissue of the prey or victim [54].

### 2.3. Minor Venom Proteins

Cysteine-rich secretory proteins (CRISP) represent a widely distributed protein family in viperid and elapid snake venoms [55,56]. Reported activities of some purified CRISPs include inhibition of cyclic nucleotide-gated ion channels and smooth muscle contraction.

The occurrence of PLB in snake venoms was initially reported by Doery and Pearson [57], and have been characterized as responsible for the high direct hemolytic activity of Australian elapid venoms by Takasaki and Tamiya [58] and Bernheimer and co-workers [59,60]. A recent shotgun proteomic analysis of the venoms of *B. atrox*, *B. jararacussu*, *B. jararaca*, *B. neuwiedi*, *B. alternatus*, and *B. cotiara*, identified PLB molecules in these six species [23], strongly suggesting that this class of toxins may be more widely distributed in Viperidae than previously thought. However, as in the case of CRISPs, the participation of this class of proteins in envenoming requires future detailed studies.

5′-nucleotidase (5′NT) was first reported by Gullan and Jackson in 1938 [61]. Since then, 5′-nucleotidase has been found in a number of snake venoms [62]. However, the pharmacological activities of this hydrolytic enzyme has not been unambiguously defined. Purines appear to be the most primitive and widespread chemical messengers in the animal and plant kingdoms [63]. The identification of free purines as endogenous constituent of venoms has further supported the role of purinergic signaling in envenomation [64]. Purines are known to potentiate venom-induced hypotension and paralysis via purine receptors. Thus, it has been proposed that, following cell disruption brought about by the venom proteinases, hemorrhagins, myotoxins and cytotoxins, purines nucleosides generated from endogenous precursors in the prey by the action of nucleotidases (5′NT, ATPase, ADPase), nucleases (phosphodiesterase (PDE), DNases, and RNases), and phosphatases (acid and alkaline phosphomonoesterases), may play role in prey immobilization [64]. In addition, ATP released from skeletal muscle by the myotoxic action of PLA_2_s acts as a “danger signal” stimulating purinergic receptors to enhance and spread the muscle damage caused by the myotoxins and pain [65,66].

### 2.4. Antivenomics

**Figure 3:**
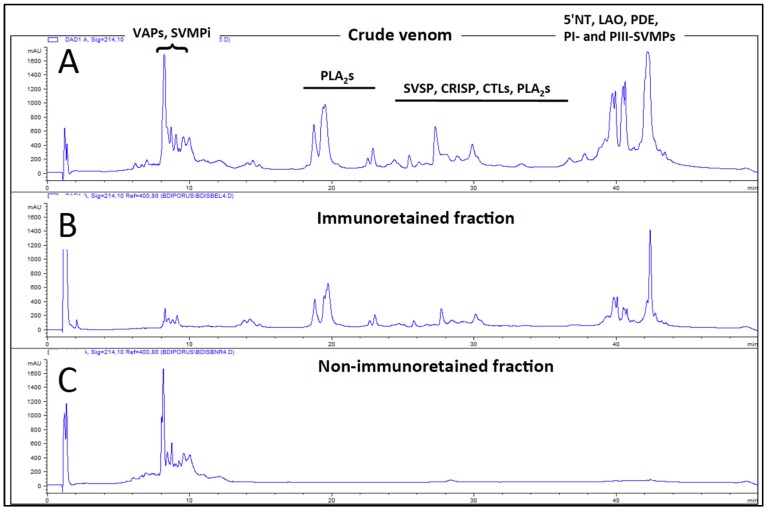
Immunocapturing ability of immobilized SAB antivenom toward the venom proteins of *B. diporus.* (**A**–**C**) show, respectively, reverse-phase separations of the whole venom components of *B. diporus*, and the immunocaptured and non-immunoretained venom proteins. Major toxin classes identified by venomic analysis (Supplementary Table S1) are highlighted in (A). Abbreviations are defined in the legend of Figure 2.

The degree of cross-immunorecognition of the venom toxins of *B. diporus* by the pentabothropic antivenom (SAB) produced by Instituto Vital Brazil was assessed by immunoaffinity antivenomics [67]. The results displayed in Figure 3 clearly show that the SAB antivenom column efficiently immunoretained all the venom components eluting in RP-HPLC fractions 12–38 and poorly those recovered in chromatographic fractions 1–11. These fractions contain endogenous tripeptide inhibitors of SVMPs (SVMPi), BPP and BPP-like peptides, and a previous investigation [49] indicated that they may not represent a serious clinical concern. On the other hand, while comparing the levels of immune recognition gathered from in antivenomics with the *in vivo* neutralization efficacy of an antivenom is not straightforward, since both experiments involve radically different protocols, in our experience, even a moderate immunocapturing capability of ~25% correlates with a good outcome in the *in vivo* neutralization tests [68]. Hence, we conclude that the SAB antivenom may represent a therapeutic alternative for treating envenomings by *B. diporus*.

## 3. Concluding Remarks

In certain *Crotalinae* genera, such as *Bothriechis*, little correlation between phylogenetic signal and venom evolvability has been reported [69,70,71,72]. However, the venomic and antivenomic analyses of *B. diporus* are in line with the reported large protein family compositional conservation across many *Bothrops* taxa, which is also reflected in their extensive cross-immunoreactivity against different antivenoms generated using different *Bothrops* venoms [20,22,23,42,73,74,75,76,77,78,79,80,81,82]. This remarkable preservation across *Bothrops* during their ~14 million years of evolution [83] points to the possibility of generating broad-spectrum bothropic antivenoms covering all the classes of medically relevant toxins.

## 4. Experimental Section

### 4.1. Venom

*B. diporus* venom was collected from 6 adult snakes kept in the Serpentarium of Corrientes, a province in Northeast Argentina. For venom extraction, the snakes were anesthetized with CO_2_. Venoms were pooled, lyophilized, and stored at −20 °C until used.

### 4.2. Isolation and Characterization of Venom Proteins

Two milligrams of crude, lyophilized venom were dissolved in 200 μL of 5% acetonitrile in water containing 0.1% trifluoroacetic acid (TFA), centrifuged to remove debris, and separated by reverse-phase HPLC using a Teknokroma Europa Protein 300 C18 (0.4 cm × 25 cm, 5 mm particle size, 300 Å pore size) column and an LC 1100 High Pressure Gradient System (Agilent Technologies, Santa Clara, CA, USA) equipped with DAD detector and micro-Auto-sampler. The flow-rate was set to 1 mL/min and the column was developed with a linear gradient of 0.1% TFA in water (solution A) and acetonitrile (solution B) using the following column elution conditions: isocratically (5% B) for 5 min, followed by 5%–25% B for 10 min, 25%–45% B for 60 min, and 45%–70% for 10 min. Protein detection was carried out at 215 nm with a reference wavelength of 400 nm. Fractions were collected manually, dried in a vacuum centrifuge (Savant), and redissolved in water, and submitted to molecular mass determination using a QTrap™ 2000 mass spectrometer (ABSciex, Concord, ON, Canada) equipped with a nanospray source (Protana, Denmark), and SDS-PAGE analysis in 12% polyacrylamide gels, under reducing and non-reducing conditions. Gels were stained with Coomassie Brilliant Blue R-250 (Sigma-Aldrich, St. Louis, MO, USA).

### 4.3. Characterization of the Venom Peptidome and Proteome

Electrophoretic protein bands were excised from a Coomassie Brilliant Blue-stained SDS-PAGE gel and subjected to in-gel reduction (10 mM dithiothreitol) and alkylation (50 mM iodoacetamide), followed by overnight sequencing-grade trypsin digestion (66 ng/μL in 25 mM ammonium bicarbonate, 10% acetonitrile; 0.25 μg/sample) in an automated processor (ProGest Protein Digestion Workstation, Genomic Solution Ltd., Cambridgeshire, UK) following the manufacturer’s instructions. Tryptic digests were dried in a SpeedVac (Savant™, Thermo Scientific Inc., West Palm Beach, FL, USA), redissolved in 15 μL of 0.1% formic acid in water, and submitted to LC-MS/MS. To this end, tryptic peptides were separated by nano-Acquity UltraPerformance LC^®^ (UPLC^®^) using BEH130 C18 (100 μm × 100 mm, 1.7 μm particle size) column in-line with a SYNAPT^®^ G2 High Definition Mass Spectrometry System (Waters Corp. Milford Massachusetts, USA). The flow rate was set to 0.6 μL/min and the column was developed with a linear gradient of 0.1% formic acid in water (solution A) and 0.1% formic acid in acetonitrile (solution B), isocratically 1% B for 1 min, followed by 1%–12% B for 1 min, 12%–40% B for 15 min, 40%–85% B for 2 min. Doubly and triply charged ions were selected for collision-induced dissociation (CID) MS/MS. Fragmentation spectra were interpreted (a) manually (*de novo* sequencing); (b) using the on-line form of the MASCOT program at http://www.matrixscience.com against the NCBI non-redundant database; and (c) processed in Waters Corporation’s ProteinLynx Global SERVER 2013 version 2.5.2. (with Expression version 2.0) against the species-specific venom gland cDNA-derived toxin sequences. MS/MS mass tolerance was set to ±0.6 Da. Carbamidomethyl cysteine and oxidation of methionine were selected as fixed and variable modifications, respectively. Amino acid sequence similarity searches were performed against the available databanks using the BLAST program implemented in the WU-BLAST2 search engine at http://www.bork.embl-heidelberg.de.

The relative abundances (expressed as percentage of the total venom proteins) of the different protein families were calculated as the ratio of the sum of the areas of the reverse-phase chromatographic peaks containing proteins from the same family to the total area of venom protein peaks in the reverse-phase chromatogram [22]. When more than one protein band was present in a reverse-phase fraction, their proportions were estimated by densitometry of Coomassie-stained SDS-polyacrylamide gels using ImageJ version 1.47 (http://rsbweb.nih.gov/ij). Conversely, the relative abundances of different proteins contained in the same SDS-PAGE band were estimated based on the relative ion intensities of the three more abundant peptide ions associated with each protein by MS/MS analysis. Finally, protein family abundances were estimated as the percentages of the total venom proteome.

### 4.4. Antivenomics

A second-generation antivenomics approach [67] was utilized to examine the paraspecific immunoreactivity of the pentabothropic antivenom produced in Instituto Vital Brazil (Niteròi, RJ, Brazil) against a pool of venoms from *B. jararaca* (50%), *B. jararacussu* (12.5%), *B. moojeni* (12.5%), *B. alternatus* (12.5%) and *B. neuwiedi* (12.5%). The final formulation consists of purified F(ab′)_2_ fragments generated by digestion with pepsin of ammonium sulphate-precipitated IgG molecules [84]. A vial of SAB (10 mL, 18.7 mg F(ab′)_2_/mL) neutralizes 65 mg of *B. jararaca* reference venom. For preparation of the antivenom affinity column, 300 μL of CNBr-activated Sepharose 4B (GE Healthcare) matrix were packed in a Pierce centrifuge column and washed extensively with 10 matrix volumes of cold 1 mM HCl followed by two matrix volumes of coupling buffer (0.2 M NaHCO_3_, 0.5 M NaCl, pH 8.3) to adjust the pH of the column to 7.0–8.0. 10 mg of antivenom were dissolved in ½ matrix volume of coupling buffer and incubated with the matrix for 4 h at room temperature. Coupling yield was 6.6 mg, as estimated by measuring the pre-coupled and post-coupled antivenom solution by UV-absorbance, using an extinction coefficient at 280 nm of 1.36 for 1 mg/mL [85]. Non-reacted matrix groups were then blocked by washing the column with 300 μL of 0.1M Tris-HCl, pH 8.0, at 4 °C overnight, using an orbital shaker. The affinity column was then washed alternately at high and low pH, with three column volumes of 0.1 M acetate containing 0.5 M NaCl, pH 4.0–5.0, and three column volumes of 0.1 M Tris-HCl, pH 8.5; this treatment was repeated 6 times. The column was then equilibrated with 5 matrix volumes of working buffer solution (20 mM phosphate buffer, 135 mM NaCl, pH 7.4; PBS). For the immunoaffinity assay, 300 μg of adult B. diporus venom were dissolved in ½ matrix volumes of PBS and incubated with the affinity matrix for 1 h at room temperature (25 °C) using an orbital shaker. This corresponded to an venom:antivenom molar ratio of about 1:22. As specificity controls, 300 μL of Sepharose 4 Fast Flow matrix, without or with 7 mg of immobilized control (naive) IgGs, were incubated with venom and the column developed in parallel to the immunoaffinity experiment. Following elution of the non-retained fractions with 2.5 volumes of PBS, the immunocaptured proteins were eluted with 5 matrix volumes of elution buffer (0.1 M glycine-HCl, pH 2.0) and neutralized with 1 M Tris-HCl, pH 9.0. The crude venom and the non-retained and the immunocaptured venom fractions were fractionated by reverse-phase HPLC using a Discovery^®^ BIO Wide Pore C_18_ (15 cm × 2.1 mm, 3 μm particle size, 300 Å pore size) column (Sigma-Aldrich, St. Louis, MO, USA) and an LC 1100 High Pressure Gradient System (Agilent Technologies, Santa Clara, CA, USA) equipped with a DAD detector. The flow rate was set to 0.4 mL/min and the column was developed with a linear gradient of 0.1% TFA in water (solution A) and 0.1% TFA in acetonitrile (solution B): isocratically (5% B) for 1 min, followed by 5%–25% B for 5 min, 25%–45% B for 35 min, and 45%–70% solution B for 5 min. Protein detection was carried out at 214 nm with a reference wavelength of 400 nm.

## Supplementary Materials

The following are available online at www.mdpi.com/2072-6651/8/1/9/s1.
